# Patient Perspectives on AI-Powered Medical Robots in Breast and Prostate Cancer Care: Qualitative Study

**DOI:** 10.2196/69710

**Published:** 2026-01-29

**Authors:** Mahiya Habib, Janet Ellis, Aaron Palachi, Melissa B Korman, Tatjana Kay, Karen Barlow, Jordana DeSouza, Rosanna Macri, Abdullah Alabousi, Mehran Anvari

**Affiliations:** 1 Department of Clinical Evaluative Sciences, Sunnybrook Health Sciences Centre, Toronto, ON, Canada; 2 Department of Psychiatry, Sunnybrook Health Science Centre, 2075 Bayview Ave, Toronto, ON, M4N 3M5, Canada, 1 416-480-6100 ext 4073; 3 Temerty Faculty of Medicine, University of Toronto, Toronto, ON, Canada; 4 Department of Psychology, Toronto Metropolitan University, Toronto, ON, Canada; 5 Centre for Surgical Invention and Innovation, Hamilton, ON, Canada; 6 Centre for Minimal Access Surgery, St. Joseph’s Healthcare, McMaster University, Hamilton, ON, Canada; 7 Department of Bioethics, Sinai Health, Toronto, ON, Canada; 8 Joint Centre for Bioethics, Dalla Lana School of Public Health, University of Toronto, Toronto, ON, Canada; 9 Department of Radiation Oncology, Temerty Faculty of Medicine, University of Toronto, Toronto, ON, Canada; 10 Department of Radiology, McMaster University, St. Joseph’s Healthcare Hamilton, Hamilton, ON, Canada; 11 Department of Surgery, Faculty of Health Sciences, McMaster University, Hamilton, ON, Canada

**Keywords:** artificial intelligence, breast and prostate cancer, cancer care, patient perspectives, patient-centered care, qualitative research, acceptability

## Abstract

**Background:**

Nearly 250,000 cancer cases are diagnosed annually in Canada, with breast and prostate cancer representing 25% and 22% of new cases, respectively. Artificial intelligence (AI) applications can potentially improve the accuracy, efficiency, and timeliness of cancer care, including screening, diagnostic imaging, and early treatment. However, patient acceptability of AI in cancer care remains underexplored.

**Objective:**

This study aimed to understand the feelings and perceptions of and acceptability to patients with breast and prostate cancer regarding the inclusion of AI-powered medical robots for cancer screening, diagnosis, and early treatment and to identify barriers and facilitators to implementation.

**Methods:**

In this qualitative study, semistructured interviews were conducted with 15 patients with breast (n=6) or prostate (n=9) cancer. Participants (mean [SD] age 67 [12] y; range 41‐88 y) were recruited from Sunnybrook Health Sciences Centre between May and November 2022. Each participant completed 2 semistructured interviews, each approximately 1 hour in length, conducted by telephone or Zoom by a research assistant. The first interview explored initial reactions and expectations regarding AI-assisted medical technologies, allowing us to tailor questions for the second interview to better understand practical means of introducing AI into care, while further exploring salient concepts. Data were analyzed using a conventional content analysis approach. Two research assistants independently and inductively coded transcripts, iteratively generating and refining a codebook. Data saturation was assessed after 10 interviews and confirmed through analysis of all 15 participants.

**Results:**

Three overarching categories were developed: (1) individual beliefs, understanding, and attitudes; (2) integration of AI into care; and (3) health structure, systems, and processes. Participants reported openness toward AI-assisted medical robots but emphasized the importance of reduced wait times, demonstrated safety and reliability, and patient-centered care. Patients indicated that with appropriate education and transparent communication, they would be willing to accept AI-assisted care due to its enhanced accuracy and efficiency. Key barriers included concerns about reliability, potential loss of human interaction, and inadequate mitigation strategies for technical failures. Facilitators included timely results, improved efficiency, accessible educational resources, and assurance that AI would complement rather than replace human expertise.

**Conclusions:**

Participants demonstrated cautious willingness to accept AI-powered medical robots in cancer care if positioned as complementary to, rather than substitutes for, human-provided care. These findings underscore the need for maintaining the presence of health care providers during AI-assisted procedures, providing clear and accessible education, and ensuring transparent communication about safety and reliability. Personalizing patient education and offering multiple modes of information delivery may foster confidence and improve acceptability. While findings are exploratory and reflect the perspectives of a small, predominantly urban sample, they provide actionable insights into patient concerns and priorities that may inform future research and guide early implementation strategies in integrating AI into cancer care pathways.

## Introduction

By the end of 2025, an estimated 248,700 new cancer cases will be diagnosed in Canada. Breast and prostate cancers are expected to account for approximately 23% of all new cancer cases [1]. For patients, receiving cancer care can be a prolonged and complicated process from screening to diagnosis and treatment [2,3]. In this article, the term *screening* is used broadly, consistent with the terminology adopted in North American cancer care research, to encompass both asymptomatic individuals undergoing high-risk surveillance (eg, breast magnetic resonance imaging (MRI) or elevated prostate-specific antigen testing) and those in the early diagnostic phase following symptom presentation or initial cancer detection. This broader operational definition reflects the continuum of early detection and diagnostic processes experienced by patients with breast or prostate cancer in the Canadian health care context. Despite the high prevalence of breast and prostate cancers, patients continue to face long diagnostic and treatment intervals, limited access to specialists, and systemic delays that negatively impact outcomes and quality of life [2-7]. Weller et al [4] refer to 3 stages of cancer care: the patient interval (symptom presentation to seeking medical attention), the diagnostic interval (seeking medical attention to obtaining a diagnosis), and the treatment interval (diagnosis to treatment) [5]. The diagnostic and treatment intervals usually include medical tests and multiple appointments with health care providers across several medical offices or hospitals [5]. Typical care pathways for prostate cancer (spanning the patient, diagnostic, and treatment intervals described by Weller et al [4]) are estimated to take between 163 and 367 days [3]. For breast cancer, the continuum from the patient interval to the completion of the treatment interval can take approximately 60 days [2]. The series of multiple diagnostic and treatment procedures contributes to long wait times and fragmented care. Such delays negatively affect quality of life, increase uncertainty, and may lead to worse health outcomes [6,7].

Timely medical attention is critical for optimal patient care, but there is a shortage of health care providers in the current Canadian health care system (ie, imaging specialists, specialized oncologists, and surgeons), which adds difficulties in meeting increasing patient demand [8,9]. Advanced-stage breast and prostate cancers may require more invasive interventions, including mastectomies and prostatectomies [10,11]. Early detection and diagnosis may allow for more targeted and less invasive treatment regimens [10,12], whereas when cancer is detected too late, less invasive options may not be possible. Across oncology and other medical specialties, health care providers experience growing workload pressures due to increasing patient volumes, staffing shortages, and other systemic inefficiencies. This has contributed to nationally elevated burnout rates and compassion fatigue among health care providers [8,9]. The limited availability and overworking of health care providers contribute to accessibility issues for patients, especially in rural and remote regions, where accessibility and availability of specialized health resources are often even more sparse [13,14].

In a systematic review, Reece et al [7] identified several key issues regarding delayed or failed follow-up for breast cancer screening in primary care settings: physician-patient miscommunication, automated alert systems creating an overinflux of information, reduced coordination of patient medical health records, and inconvenient clinic hours and lack of availability of primary care. Urquhart et al [15] interviewed survivors of prostate cancer about their experiences with follow-up and posttreatment cancer care. Participants described various issues, including lack of information, resources, and psychosocial support, which left them feeling unprepared in their recovery process. These perceived issues in their care were related to lack of availability of health care providers and not being able to see the same health care provider throughout their care due to a staffing shortage within oncology [8,15].

Recent advances in technology, such as the use of artificial intelligence (AI) in cancer screening, represent a potential mechanism for increasing efficiency in wait times in cancer pathways, reducing the demand on health care providers, and improving accessibility within the health care system [16,17]. AI has been shown to be an effective tool for breast and prostate cancer diagnosis and in prostate cancer Gleason scoring [18]. Successful AI applications in diagnostic imaging have enabled the detection of early cancerous lesions with greater accuracy and efficiency, with trialed technology often outperforming experts [19,20]. A 2017 study showed that automated deep learning algorithms identified nodal metastases in histopathological analyses of breast tissue more accurately than an expert panel of pathologists [21]. AI deep learning algorithms have also assessed mammograms with proficiency equal to expert radiologists, with 5.7% and 9.4% reduction in false positive and false negative rates, respectively [22]. In prostate cancer screening, deep learning algorithms have demonstrated the potential to automate Gleason scoring of histopathologic images of adenocarcinomas, achieving 75% agreement with expert pathologists, demonstrating great model performance [18]. Similar AI-powered applications have been used with diffusion-weighted MRI to delineate cancerous and noncancerous prostate tissue [23]. As AI becomes more sophisticated, new opportunities in both diagnostic imaging and treatments are emerging with potential to transcend the accuracy and time limitations of traditional methods. Specifically, with the advent of AI and robotics, telemedicine applications are feasible and have the potential to mobilize high-quality health care, allowing interventions to be carried out by medical robots, without a highly trained specialist onsite [18,23].

While AI-powered robotic systems have the potential to expedite procedures and improve patient care, the success of these technologies is contingent on patient acceptance. The needs and preferences of patients should influence both the development and the process of implementation of such technology into the health care system. This requires a comprehensive understanding of the patients’ perceptions toward the use of such technology in their care. A greater understanding of what may be deemed acceptable is necessary before conducting large-scale research evaluating the use of AI in breast and prostate cancer screening and treatment [24,25]. The present study sought to elucidate patient perceptions, feelings, and acceptability regarding AI-powered medical robots in breast and prostate cancer care and to identify barriers and facilitators to implementation using a person-centered approach.

## Methods

### Study Design

This study employed a qualitative descriptive approach to answer the research question: How do patients feel about the use of AI-powered medical robots for cancer screening, diagnosis, and early treatment? Study objectives were to (1) illuminate patient understandings and feelings regarding the inclusion of AI in their care and (2) identify barriers and facilitators of implementing AI-powered robotic systems into cancer screening from patients’ perspectives. This study is reported in accordance with the COREQ (Consolidated Criteria for Reporting Qualitative Research) guidelines (Checklist 1) [26]. A postpositivist stance [27] was taken, which assumes that while absolute truth cannot be fully captured, systematic qualitative methods can approximate patient perspectives and generate transferable insights. This orientation informed our design by emphasizing structured data collection (semistructured interviews), analytic rigor (systematic coding by multiple analysts), and reflexivity to minimize bias. This study was part of a larger program of work initiated by a multidisciplinary research team developing and testing AI technology for cancer care. Although not embedded within a larger trial, it was designed to inform future research protocols and ethical considerations for implementing AI-powered medical robots. The overarching program sought to explore the feasibility of systems capable of scanning, diagnosing, and treating patients within a single appointment. This qualitative study, therefore, focused on understanding patient comfort and acceptability of such technologies across the cancer care continuum, including perspectives on initiating treatment immediately following diagnosis.

### Participants

Participants were recruited from breast and prostate cancer clinics at the Odette Cancer Centre at Sunnybrook Health Sciences Centre between May 2022 and November 2022. Purposive sampling was used to identify individuals at elevated risk of breast or prostate cancer, or with a recent diagnosis, to ensure that perspectives from both patient groups were represented. Potential participants were first identified by their treating physicians, who requested permission for research staff to contact them. Research staff approached participants by telephone following referral, explained the purpose of the study, and assessed their eligibility and interest. Inclusion criteria were (1) English speaking, (2) aged 18+ years, and (3) patients deemed *high risk* by their physician and in need of MRI as part of their screening process for breast cancer or diagnosed with breast cancer *or* male patients with elevated prostate-specific antigen levels and considered *high risk* for prostate cancer or diagnosed with prostate cancer.

### Ethical Considerations

This study was reviewed and approved by the Research Ethics Board at Sunnybrook Health Sciences Centre (Research Ethics Board 5361). All participants provided verbal informed consent prior to participation, which was documented by the study team in accordance with institutional guidelines. To protect privacy and confidentiality, interview transcripts were deidentified during the transcription. All data were stored securely on password-protected institutional servers with access restricted to the study team. Participants were compensated with CAD $50 (US $36.03) per interview session, in recognition of their time and contribution.

### Data Collection

Demographic information including sex and gender, ethnic identity, socioeconomic status, and geographic data (rural or urban) was collected prior to the first interview. While participants did report gender, they were referred based on a biological basis (ie, risk for or current diagnosis of prostate or breast cancer). Two semistructured interview sessions, each approximately 1 hour in length, were conducted over telephone or video (Zoom) by a graduate-level research assistant (TK) who had been trained in qualitative interviewing, with a background in clinical psychology, psychology, global health, and sociology. The 2 interviews were conducted approximately 2 weeks apart, allowing time for reflection and deeper meaning-making while maintaining recall continuity [28,29].

An interview guide was used, with flexibility for the interviewer to probe participants’ experiences and perspectives. The interview guide included open-ended questions about participants’ general perceptions of AI in health care; specific feelings about AI-powered robots in cancer screening, diagnosis, and treatment; perceived barriers and facilitators to AI adoption; and preferred communication/education strategies. The full interview guide is provided in Multimedia Appendix 1. During interviews, the interviewer clarified that the term *AI-powered medical robots* referred broadly to robotic systems enhanced with AI that could be applied to different parts of the cancer pathway, including imaging, biopsy guidance, and treatment support. This clarification was intended to provide participants with a shared reference point, while still allowing their natural perceptions and assumptions to guide their responses. Participants were not given a detailed technical briefing, as the intent was to elicit their existing awareness and spontaneous perceptions of AI technologies in health care. This approach ensured that participant reflections represented authentic, experience-based understandings rather than those influenced by study-provided information.

All participants were informed during recruitment and reminded prior to each interview that the study focused on their cancer care experiences and their perspectives regarding AI-powered medical robots. The first interview emphasized participants’ initial reactions, beliefs, and expectations about AI-powered medical robots in cancer care. The second interview revisited these themes in more depth, provided an opportunity to clarify or expand on responses, and explored additional reflections participants had after the first discussion. This consistent framing ensured that discussions remained grounded in participants’ personal experiences of cancer care while allowing flexibility for individual interpretation and reflection.

### Data Analysis

Interview recordings were transcribed verbatim by 2 research assistants (MH and JD). A conventional content analysis approach, which relies on inductive coding [30], was followed. Coders (1) immersed themselves in the transcripts, (2) performed line-by-line coding, (3) grouped codes into subcategories, and (4) clustered subcategories into broader categories. The codebook (Multimedia Appendix 2) was developed inductively from the data, with new codes added when concepts not captured by existing codes were identified. Iterative updates to the codebook were justified when coders encountered new or refined meanings across interviews, and changes were documented in the audit trail. To ensure rigor, MH and JD independently coded 30% of the transcripts, meeting to compare and reconcile code discrepancies. Disagreements were resolved through consultation with a third research team member (MBK), a graduate-level research coordinator with training in qualitative research methodology. Once agreement on the initial codebook was achieved, the coders each analyzed half of the remaining transcripts, routinely consulting with one another and MBK to discuss emerging ideas and refine codes as needed. The final coding tree was established by the 3 reviewers with input from a fourth member of the research team (AP) and the site principal investigator (JE). Including multiple perspectives helped limit the potential impact of biases of any given researcher.

Though formal coding began after all interviews were completed, the interviewer made informal notes on emerging themes during the interviews to assess data saturation [31] and guide iterative refinement of the interview guide. Reflexive memos were maintained to document thoughts on the saturation process. Reviewers maintained a thorough audit trail outlining the identification and adaptation of new and existing codes. Saturation was then formally assessed retrospectively, over the course of data analysis. Coders reviewed transcripts sequentially and confirmed that after the tenth interview, no new substantive codes were identified. The remaining interviews contributed additional depth and nuance but did not generate new categories, confirming that saturation had been reached. Given that participants were recruited from a single large urban tertiary cancer center and the sample was predominantly White and urban-residing, it is possible that this relative demographic homogeneity contributed to the point at which saturation was reached. This demographic profile reflects the typical patient population served within the breast and prostate cancer clinics at Sunnybrook Health Sciences Centre.

### Positionality Statement

The research team represented a range of disciplinary, cultural, and experiential backgrounds. Interviews were conducted by a female master’s student and research assistant with training in clinical psychology, global health, psychology, and sociology. Coding and analysis were carried out by 2 female research assistants (BAs in psychology), a female research coordinator with a master’s degree in medical sciences and formal qualitative research training, and a male research coordinator with a BA in psychology and training in clinical psychology. Team members reflected diversity in gender and cultural backgrounds, which contributed to varied perspectives in the analytic process. The site principal investigator, a female psychiatrist specializing in psychosocial oncology and trauma, and the coprincipal investigator, a male professor of surgery with expertise in intelligent robotics, provided oversight for the study.

### Trustworthiness

To mitigate potential bias and increase credibility of the findings generated in this study, we used reflexive practices, including regular peer debriefing between the coders and the third reviewer, to identify, acknowledge, and reconcile assumptions and interpretations. Engaging in reflexivity as we continuously rereviewed and discussed the transcripts allowed us to ensure that patient voices remained central in the findings, which supported confirmability of the findings. We also transcribed audio recordings verbatim for analysis, kept a thorough audit trail, and have thoroughly contextualized our work and described our methods as means of increasing dependability of this work. To address transferability, we have provided a thorough description of the study methods (including the interview questions) and the participant demographics.

## Results

### Participant Demographics

In total, 15 participants (6 female and 9 male participants) were recruited, all of whom completed both interviews. Of the 14 participants who reported demographic data (Table 1), 80% (n=12) were White. The mean age of participants was 67 (SD 12; range 41‐88) years. 80% (n=12, 7 male and 5 female participants) of reporting participants lived in urban settings, 13% (n=2, 1 male and 1 female participant) lived in rural settings. Of the 10 participants who reported their annual household income, 7 (70%) reported an annual household income over US $60,000.

**Table 1.** Participant demographics^a^.

Demographic category	Count, n (%)
Sex	
Male	9 (60.0)
Female	6 (40.0)
Ethnic identity	
White/Caucasian	12 (80.0)
Asian	1 (6.7)
Middle Eastern	1 (6.7)
Age range (y)	
40‐49	1 (6.7)
50‐59	2 (13.3)
60‐69	5 (33.3)
70‐79	5 (33.3)
80‐89	1 (6.7)
Household income^b^ (US $)	
Below 14,630 (CAD 20,000)	1 (6.7)
14,630 (CAD 20,000) - 29,258‐( CAD 39,999)	2 (13.3)
29,259 (CAD 40,000) - 43,888 ‐(CAD 59,999)	—^c^
43,889 (CAD 60,000) - 58,517 ‐(CAD 79,999)	4 (26.7)
58,518 (CAD 80,000) - 72,416‐(CAD 99,000)	—
73,148+ (CAD 100,000+)	3 (20.0)

a One prostate cancer participant did not report any demographic information.

b Household income was originally collected in Canadian dollars and converted to US dollars using a currency exchange rate of CAD $1 = US $0.73

c Not available.

### Thematic Findings

**Figure 1.**
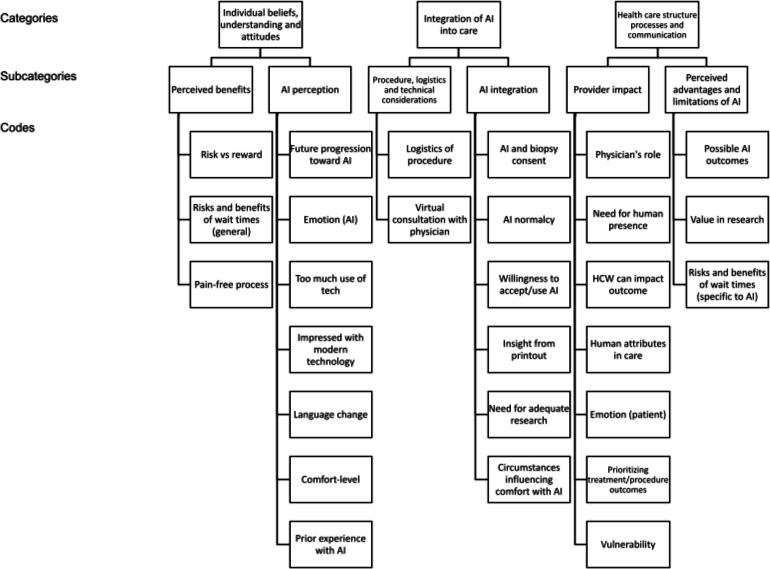
Final coding tree: organization of codes>subcategories>categories. AI: artificial intelligence; HCW: health care worker.

The analysis yielded 28 codes, which were then organized into 6 subcategories and further grouped into 3 overarching categories to best understand participant feelings of integrating AI-assisted medical technologies into their cancer care. The 3 overarching categories were (1) individual beliefs, (2) understanding, and (3) attitudes, integration of AI into care, and health care structure, process, and communication (Figure 1).

In the *individual beliefs, understanding, and attitudes* category, participants described a mix of openness and hesitancy toward AI-assisted technologies in their cancer care. Acceptability often depended on whether AI could meaningfully improve the experience of care, such as offering greater accuracy, reducing discomfort, or improving efficiency. Ongoing concerns centered on overreliance on technology, safety, and the need for clear backup plans in case of system failure. While some participants negatively described a general overreliance on technology, others noted that acceptance would increase as evidence accumulated:

*[T]hey [are] as good as a human.*

[ID 15, F, 53]

Concerns about dependability were especially salient in the context of unexpected events:

*[C]ertain things can happen, who would be available, in an electrical shutdown and so on? [...] I don't know the capability of these machines, how reliable they are [...] it’s reassuring to have help nearby to jump in.*

[ID 50, M, 73]

Across participants, trust in AI was described as something that would grow through experience and familiarity.

The *Integration of AI into Care* category focused on the procedural, technical, and logistical considerations of integrating AI-robotic medical technology into cancer care, as well as general perceptions on AI integration into care. Participants distinguished between different stages and levels of invasiveness in the cancer pathway. Many were comfortable with AI in roles such as imaging or screening but more cautious regarding AI involvement in biopsies or surgery.

As one participant summarized:

*I think I am okay with [the machine doing] the screening, if you could speed up the wait time. For the actual surgery I think I need more proven evidence, that it is 100% positive and reliable.*

[ID 30, F, 62]

Human presence, whether in-person or virtual, was consistently described as important for reassurance, oversight, and troubleshooting during procedures:

*[K]nowing that they were there and that there was some sort of troubleshooting capacity, would be helpful.*

[ID 80, M, 65]

Participants also highlighted the need for accessible explanations of how AI works, its benefits, and its limitations. They emphasized that information should be made available proactively and through multiple accessible formats, given that patients may be unfamiliar or uncomfortable with digital resources:

*I had to take the initiative to search through the website to get [information] and knowing so many people, they are not familiar with using technology, or some seniors, I think that was, you know, it limited the opportunity for people to get [access to] support resources.*

[ID10, M, 60]

The category *health care structure, processes, and communication* described challenges within the existing health care system, particularly long wait times, fragmented communication, and limited opportunities for personalization. These issues shaped patients’ broader care experiences and influenced how they envisioned AI fitting into their care. Some felt “lost in the system” [ID 45, F, 69], due to the perception that the current health care system lacks personalization.

Participants also associated long delays and fragmented care with distress and uncertainty:

*I couldn't get any surgery done immediately. [...] It was very difficult. And I couldn’t get care in a timely way.*

[ID 40, M, 78]

Several participants expressed hope that AI-assisted technologies might address some of these system-level pressures by supporting more timely and/or coordinated care, while emphasizing that technological integration should not reduce human connection in the care process.

### Barriers and Facilitators

Across interviews, participants described specific conditions that shaped how acceptable AI-assisted medical technologies felt to them in their care. Consistent with qualitative studies examining patient adoption of new health technologies and with implementation science frameworks (eg, studies that explore contextual factors influencing uptake) [31], these perceived conditions are referred to as *barriers* (factors that reduced comfort or confidence) and *facilitators* (factors that increased openness or ease of acceptance). Table 2 provides an overview of the barriers to integrating AI-assisted medical robots into cancer screening, diagnosis, and treatment.

**Table 2.** Factors influencing patients’ decision to accept AI^a^ in cancer care (barriers and facilitators).

Factor	Barrier	Facilitator
Information	Lack of information regarding the technology left patients unable to answer whether they were comfortable using it.	Providing adequate patient education and related resources would facilitate the uptake of AI-assisted technology.
Vulnerability	These patient populations are particularly in need of human care due to the vulnerable nature of the procedures.	—^b^
Reliability of technology	Fear of technical malfunctions or similar issues.	Potential for increased precision and efficiency with the use of technology; some patients noted that the most important thing is a positive outcome, regardless of whether a doctor or robotic device is completing the procedure.
Human element	Machine lacking in compassion/bedside manner in critical thinking/problem-solving skills.	No risk of human error when using AI technology.
Reduced wait times	—	The potential for reduced time between initial appointment and treatment can improve patient outcomes and eliminate distress associated with waiting periods.
Prior use of AI-assisted technology in health care	—	Patients express prior knowledge or direct experience with some form of AI technology in health care, creating a greater sense of comfort with using AI.
Pain	—	Some participants stated that they would be more inclined to consent to the procedures if they were less painful than the standard of care.
Options available	Patients might prefer standard of care when they have the option (given that the risks/rewards are similar between these options).	If there were no other options, or standard of care came with increased risk (due to waiting times, etc), patients would be more likely to consent to use of AI-assisted technology.

a AI: artificial intelligence.

b Not applicable.

Barriers most often reflected concerns about the interpersonal and safety-related dimensions of AI-assisted care. Participants frequently worried about the absence of human qualities such as compassion, empathy, and emotional support, particularly during invasive or high-stakes procedures. Many also questioned the reliability and safety of AI systems, describing uncertainty about how unexpected complications would be managed without a health care provider physically present. Some participants expressed apprehension that technological integration could reduce opportunities for communication with clinicians, thereby amplifying existing feelings of depersonalization within the health care system.

Facilitators described by patients centered on ways AI-assisted technologies could meaningfully improve their care experience. The potential to reduce wait times, streamline care, and deliver more timely results was consistently valued and often linked to reductions in distress, uncertainty, and logistical burden. Participants also described their confidence in AI’s technical capabilities, such as accuracy and precision, as an important factor that could support acceptance. Several participants noted that when outcomes were perceived as likely to improve, the mode of delivery (human vs robot) became less central. Prior familiarity with AI in health care and the possibility of less painful or less invasive procedures further contributed to openness toward AI integration.

## Discussion

### Principal Findings

This study found that patients with breast and prostate cancer expressed cautious openness toward the use of AI-powered medical robots in their care, despite initial skepticism. Participants recognized potential benefits such as reduced wait times, improved accuracy, and less invasive procedures and also voiced concerns about safety, reliability, and the potential loss of human connection in care. Participants expressed enthusiasm for AI-assisted medical robots when given the choice, suggesting openness to innovation despite some skepticism. They often prioritized successful treatment outcomes over concerns of who or what delivered care, indicating that demonstrated efficacy could outweigh reservations. Trust in AI was described as something that would build gradually over time as experience and familiarity increased. Their accounts also underscored the critical need for systemic improvements in health care processes, particularly in reducing delays and streamlining care. These findings highlight the perceived potential of AI technologies to address longstanding system challenges, while also reflecting ambivalence regarding the risks of technological reliance. Notably, participants were generally open to the use of AI-powered medical robots in initial screening and imaging but were more hesitant when considering invasive steps such as biopsy or treatment, where human presence and oversight were prioritized.

As an exploratory qualitative study, the aim was to elucidate patients’ feelings, understandings, and perceptions about the use of AI-powered medical robots in cancer screening, diagnosis, and early treatment and to identify barriers and facilitators to patient acceptance. Participant responses illustrated a mix of hesitancy and hope around embracing this technology for their own care, which is a typical response to technological advances or change [30,31]. Importantly, participants’ reflections consistently emphasized that the human presence in care—particularly the compassion, reassurance, and emotional support provided by health care providers—is viewed as irreplaceable [32]. This emphasis on the importance and value of human presence in care (particularly that health care providers should remain central in a patient’s care processes) underscores the importance of emotional support and other traits that are unique to humans. Without deliberate safeguards to preserve these aspects, the integration of AI technologies risks exacerbating feelings of depersonalization in care. These insights, while not generalizable due to the small purposive sample, provide valuable direction for future research and early implementation strategies.

### Comparison to Prior Work

Overall, participants’ reflections were broadly consistent with existing research examining patient perspectives on AI and technology-assisted care. Similar to prior work, participants expressed interest in potential benefits such as improved efficiency and reduced wait times. These factors are repeatedly identified in the literature as persistent challenges in cancer care pathways [7,16,17]. Our findings align with studies showing that patients often view AI as promising when it addresses established system-level pain points, particularly diagnostic delays and fragmented processes.

Concerns regarding the loss of human connection were also consistent with previous research demonstrating resistance to medical AI when patients perceive threats to empathy, communication, or relational aspects of care [33]. Participants emphasized the irreplaceable value of compassion and reassurance, echoing patient-centered care literature, highlighting communication, emotional support, and trust as core needs during cancer care [34-36]. Prior studies similarly note that while AI may assist with technical tasks, it cannot fully replicate relational qualities valued in clinician—patient interactions [32,33].

Consistent with earlier work, participants indicated that evidence of safety and effectiveness would be central to their acceptance of AI-assisted technologies. This is in line with studies showing that patient trust in AI increases when technologies are validated, transparent, and clearly integrated within human-led care models [30,31]. The desire for clear education about AI, expressed by many participants, is also supported by previous findings emphasizing the importance of accessible, multimodal patient education to support uptake of new technologies [35,37,38]. Altogether, the results reinforce themes seen across the broader literature: patients may be cautiously optimistic about AI in cancer care when it enhances, rather than replaces, human-delivered care; when it improves timeliness and coordination; and when adequate evidence and communication support informed decision-making. These consistencies suggest that early implementation strategies should prioritize transparency, patient education, and explicit preservation of human presence, particularly during invasive or emotionally charged stages of cancer care.

### Strengths and Limitations

There are several limitations to this study. First, recruitment occurred at a single large urban hospital, which may not be representative of all patient populations. Participants were drawn from a tertiary cancer center in Toronto, potentially excluding individuals from rural or underserved regions and therefore excluding perspectives from those with limited access to tertiary care. This is related to a previously established limitation regarding the disparity in who has access to high-quality health care [39]. This may have influenced findings by overrepresenting patients with greater access to resources and technology. Future research should intentionally recruit participants from rural and underserved regions to increase transferability.

Second, the sample was predominantly White (80%), urban-dwelling, and higher-income. Urban and higher-income populations may have more exposure to and comfort with advanced technologies compared to their rural and lower-income counterparts [40]. Additionally, the perspectives of those who live in rural settings may be impacted by experiences with resource limitations and accessibility of health care [41,42]. As a result, the findings may not reflect the views of patients from more diverse racial, ethnic, or socioeconomic groups. Within the constraints of this study setting and recruitment pathways, we were unable to broaden the sample to include more diverse participants. Although participants were provided with a shared reference point for what was meant by “AI-powered medical robots” (ie, robotic systems enhanced with AI used in imaging, biopsy guidance, and treatment support), they were not given a detailed technical briefing. As such, their responses may have reflected variability in baseline knowledge. Some participant responses also appeared general in tone (eg, referring broadly to *AI* or *technology*), but these were expressed in relation to their cancer care experiences. This likely reflects natural variability in familiarity with AI concepts among participants rather than deviation from the study focus. This could be seen as a limitation in terms of consistency, but it also provided valuable insights into organic perceptions, assumptions, and information needs regarding AI in cancer care. Third, data collection took place during the COVID-19 pandemic, which may have impacted attitudes toward access to health care, or experiences with health care delays may have been impacted by the health care climate at the time [43,44]. This context likely heightened sensitivity to wait times and system inefficiencies. While this provided timely insights, future studies should examine whether similar themes emerge in nonpandemic contexts. Finally, the sample size was relatively small, though appropriate for a qualitative descriptive study. Data saturation was achieved and confirmed during analysis; however, larger samples in future research could support broader transferability of findings and capture a greater diversity of perspectives. Despite its size, the study’s use of 2 interviews per participant and a multianalyst coding process strengthened the dependability and credibility of the findings. Additionally, collecting perspectives from both breast and prostate cancer populations provided a richer understanding of how AI-assisted technologies may be perceived across different cancer care experiences.

### Future Directions

Future research should ensure to include perspectives from patients who represent more diverse lenses as a means of increasing transferability of findings and understanding specific needs for integrating AI-assisted medical technology into cancer care in varying regions and cultural contexts. Future studies should also aim to include perspectives from all involved in the health care process (such as physicians and oncology assistants) to better understand how to pragmatically incorporate AI-assisted medical robots into cancer care screening, diagnosis, and treatment. More work is needed to understand which implementation models work best for both health care providers and patients, including how to provide education on the technology to both, what level of human presence is deemed acceptable and appropriate, and safe mitigation strategies in the event of technical issues. Future trials should also examine patient acceptability separately across different stages of the cancer pathway (eg, screening, diagnosis, and treatment) to determine where AI integration is most feasible and where stronger human involvement is needed.

### Conclusions

In conclusion, this work explored patient perspectives on the use of AI-assisted medical robots in cancer screening, diagnosis, and early treatment, highlighting barriers and facilitators to its acceptance. The findings reveal a cautious optimism among patients, tempered by concerns about the reliability of AI and the potential for reduced human contact. Patients expressed a desire for AI to complement, rather than replace, human judgment, ensuring that final decisions remain in the hands of experienced health care providers. Alongside emphasizing the need for human presence and emotional support within the health care experience, patients described a need for education and clear communication regarding the use of new technology and suggested potential methods of making these resources more accessible (eg, through media and information sessions). As a qualitative exploratory study, these conclusions should be understood as reflecting patient perceptions within the sampled group, rather than definitive statements about broader populations. Future research is required to test these perspectives in larger and more diverse samples and to evaluate whether these patient-identified barriers and facilitators translate into measurable outcomes during actual implementation.

## Supplementary material

10.2196/69710 Multimedia Appendix 1 Interview guide.

10.2196/69710 Multimedia Appendix 2 Codes and definitions.

10.2196/69710 Checklist 1 Completed COREQ checklist.

## Abbreviations

AI: artificial intelligence
COREQ: Consolidated Criteria for Reporting Qualitative Research
MRI: magnetic resonance imaging
