# Supplementary material for: Patient Perspectives on AI-Powered Medical Robots in Breast and Prostate Cancer Care: Qualitative Study
Source: JMIR Cancer. 2026 Jan 29;12:e69710. doi: 10.2196/69710 (PMC12854401; doi:10.2196/69710)
Supplement: Multimedia Appendix 1 [file cancer-v12-e69710-s001.docx]

Interview Guide

**Questions for First Interview**

Introduction: "Thank you for being here today. You've been invited either as someone who has been treated for breast/prostate cancer in the past, or whose clinician has recommended that you undergo MRI imaging to screen for potential lesions.

I want to note that we will be recording the session today, but this recording will be destroyed after it is transcribed, and the transcription will be de-identified so that your name or identifying information is not associated with it. Additionally, any papers or reports associated with this work will not have any identifying information. We ask that you help us to protect anonymity by keeping the experiences shared here private.

Our goal in this interview is to explore how patients feel about the potential use of artificial intelligence-powered medical robots for cancer screening, diagnosis, and early treatment. Artificial Intelligence refers to the use of a computer to control a robot that can perform tasks normally conducted by a human. This has many potential medical applications, including for cancer diagnosis and treatment where a robot can perform procedures. We will now head into our questions. Please share whatever comes to mind, that you are comfortable sharing.

Do you have any questions or concerns before we begin?"

1. ​How do you feel about the use of AI technology in cancer screening and diagnosis?

For the next set of questions, we want you to imagine that we are living in a future world, where AI-assisted cancer screening, diagnostics and some treatments have been proven to be effective, accepted by regulatory bodies, and are used routinely in cancer care.

1. What would you need to know about the process of having AI involved in cancer screening and the process of diagnosis, to decide whether you would be comfortable using this form of technology?
   1. How would you feel about the robot conducting interventional procedures, such as a biopsy, rather than an oncologist or radiologist?
   2. Do you have any concerns about the use of AI in cancer care?
2. Without AI technology patients undergo an MRI and if a suspicious lesion is detected they then may be required to have a biopsy done. An AI-assisted medical robot would be able to interpret your MRI to identify whether suspicious lesions are present, and if deemed necessary based on the AI-informed reading as well as confirmation from a radiologist or oncologist, conduct a biopsy. How would you feel about allowing an AI robot to do this? We should note that a technician would be present.
   1. How would you feel about the physician (radiologist or oncologist) consulting with the technician virtually to confirm the readings, rather than being there in person?
   2. Would you be comfortable consenting to a biopsy prior to the MRI, so that the biopsy can proceed right away if there was a suspicious lesion identified?
   3. How would you want to be informed that there is a suspicious lesion, and that the biopsy is going to happen? Could the machine tell you, or would it need to be a human?
3. If you did go ahead with the biopsy, and the AI-assisted device was able to conduct histopathology in order to make a diagnosis, how would you want to receive those results?
   1. Future appt with physician?
   2. Immediately from the technician? **Assuming that this is legally allowed in future** What if a physician was there virtually?
   3. Printed readout from the machine along with the chance to discuss with the technician or other clinicians?
4. FOR PROSTATE PATIENTS ASK: if it were possible to begin treatment right after diagnosis, how would you feel about having the treatment immediately after diagnosis by the same medical robot that conducted the biopsy?​

Before we end off today, we have a few final questions to gather your overall insight on the proposed process of AI-assisted cancer screening and diagnosis.

1. What might you want the team developing this type of technology to know? It could relate to your experience with cancer care, diagnosis and treatment, general thoughts on the proposed technology, things to consider during the process, or anything else that comes to mind.
   1. Do you have any recommendations for ways to make the process more acceptable to and comfortable for patients?
   2. Are there any supportive resources you'd recommend making available to patients to support those experiencing diagnosis and treatment using AI medical robot technology? Webinars, Q&A sessions with staff, etc?

Before we finish off today, do you have any final thoughts or concerns that you haven't had the chance to share yet?

**Second (Follow-Up) Interview**

1. How do you feel about the potential use of this form of AI-assisted medical robot in cancer screening and diagnosis?
   1. As this technology could speed up the process of receiving diagnostic information (rather than waiting days-to-weeks between MRI, biopsy, and receiving results, you could receive them immediately), how would you feel about receiving the biopsy results immediately, according to the AI histopathology?
2. What else would you need to know about the process during consent, to make an informed decision regarding your care?
   1. How do you feel about a "stepped" consent process, where you can choose to consent to just MRI, vs MRI with possible biopsy, etc. prior to beginning with the MRI? Or would you prefer to consent to each step after finishing the last one?
3. Assuming clinical trials were successful, how likely would you be to consent to using a medical robot in your cancer care?
4. Do you have any final thoughts that you would like to share with the group, or for us to share with the tech developers?
