# Supplementary material for: Patient Perspectives on AI-Powered Medical Robots in Breast and Prostate Cancer Care: Qualitative Study
Source: JMIR Cancer. 2026 Jan 29;12:e69710. doi: 10.2196/69710 (PMC12854401; doi:10.2196/69710)
Supplement: Multimedia Appendix 2 [file cancer-v12-e69710-s002.docx]

Appendix C. Codes and Definitions

| **Code** | **Definition** | **Participant Quotes** |
| --- | --- | --- |
| **Need/preference for reduced wait times** | Patient emphasizes that cancer care must prioritize being quick and efficient | “I would want to begin treatment as fast as possible, [...] that’s the main thing. If you have to wait and wait and wait and then that’s not good.” (ID 60, M, 86) |
| **Risks and benefits of wait times (general)** | Patients share thoughts and experiences related to delays in their care experience | “The important things we need is faster, accurate diagnoses and availability is crucial and even if people are super affable it's no good if you can't get care.” (ID 50, M, 73) |
| **Risks and benefits of wait times (specific to AI)** | AI influence on treatment wait times | “You’ve got to say that ‘we have a fantastic new process which will reduce your waiting time, reduce your visits and it's well proven and we were very impressed by it’ That will make people want it.” (ID 25, F, 40) |
| **Systemic issues** | Need for systemic change | “I think I see a lot of people get lost in the shuffle in our medical system - they can't make contact with doctors or they just get lost. We need to improve things.” (ID 15, F, 53) |
| **Comfort-level** | How comfortable patients are with AI and receiving cancer care from AI | “My comfort would depend on what’s happening, what stage and everything going on. If I feel connected to everyone and the processes going on, I will feel more comfortable. But it depends. Of course in any sort of procedure I would still need a person to be comfortable.” (ID 65, F, 53) |
| **Future progression towards AI** | Sentiments of how ultimately society is moving toward more tech/AI | “I think it's a very inevitable direction for this type of medical diagnostic to go, because I think AI is definitely ultimately going to be in charge of these types of sort of reading of data from instruments like MRI's, I mean it's just the machines are better at it. That’s just the way it is.” (ID 30, M, 62) |
| **AI and biopsy consent** | Thoughts and feelings related to AI doing biopsies | “I: Would you be comfortable consenting to a biopsy prior to the MRI's so that the biopsy can proceed right away if there was a suspicious lesion detected? P: That doesn't bother me, no. I: And how would you want to be informed that there was a suspicious lesion and that the biopsy was going to happen? P: That has to be a human, because now we’re at a different level. The machine can’t do that.” (ID 55, F, 65) |
| **Physician's role** | Patient expresses importance/need for physician | “Talking to a doctor during diagnostics is important. The approach of human beings when they're saying these technical and sensitive things, I'm not quite sure if a robot can do that but obviously, when they do the actual procedure, that is different than the delivery.” (ID 10, M, 60) |
| **Emotion (AI)** | Patient shares their thoughts on emotion in relation to AI (e.g., AI capacity to show emotion) | “Very simply, I still think that medicine has as much art in it as it has science and there is no art in a robot.” (ID 15, F, 53) |
| **Emotion (patient)** | Patients share that their emotions/the emotional aspect of care needs to be maintained | “If I did go ahead with the biopsy and the AI assisted device was able to conduct the histopathology in order to make a diagnosis, how I’d want to get my results back connects to the emotional aspect. If the technician was capable and emotionally physically – emotionally capable also to pass on the information [about the diagnosis], he could say ‘we found something, but it doesn't seem to be too serious; however, we will let you talk to a doctor.’ Rather than say ‘we found something, better see a doctor.’ Again, that's where the emotional aspect comes into it again. You gotta make that person feel comfortable.” (ID 10, M, 60) |
| **Human attributes in care** | Patients share what attributes they deem to be important in care | “One of the main things in the medical field is having kindness and compassion, which a computer can't do.” (ID 45, F, 69) |
| **Patient care experience** | Perspectives, feelings, and preferences of patients regarding various aspects of their cancer care experience, and factors influencing their perspectives in using AI | “There were a lot of decisions that I had to make and the doctor explained to me that, you could do this, or this, or this, and he recommended which one to do and I approved it. I said okay, let’s go with this. This helped me feel like I had control and was part of it.” (ID 35, F, 75) |
| **Need for human presence** | There needs to be a person involved in the process to reassure, doesn’t necessarily need to be a doctor | “The process had yet to have any true success yet but there's been lots of contact and lots of coaching and lots of human intervention in the process and that's appreciated.” (ID 20, M, 77) |
| **Need for fast and accurate diagnoses** | Participant expresses that it's important to make sure that there are fast and accurate diagnoses given | “My main recommendation and concern is getting it as fast as possible. That’s the main thing.” (ID 40, M, 78) |
| **AI Normalcy** | Patient speaks to the fact that since AI is a new technology in healthcare, it will take time for the public to get used it | “It's reassuring to have a physician on standby at least until things have been proven with more experience.” (ID 65, F, 53) |
| **Educational resources** | Educational information and resources desired | “To feel comfortable, really any information that was already available or places to look for information were key. It's better having access to resources. Having someone to walk you through the consent but then having the resources available and if you need help, and having that available if they wanted it.” (ID 10, M, 60) |
| **Circumstances influencing comfort with AI** | Patient expresses situations and circumstances where they'd be comfortable or uncomfortable with AI | “ If [the procedure] was pretty straightforward, I would probably be comfortable. If [the procedure] was awkward, I’d be a little bit more leery [...] it would depend on the circumstance.” (ID 45, F, 69) |
| **Desire for information** | Information required about AI | “I would want to know what the process is with the artificial intelligence as to exactly what's going to be done, how it will be used.” (ID 50, M, 73) |
| **Need for adequate research** | Need for established research in development and performance of AI | “If it's still just in trials I'd have to think a lot harder about how much permission I would be happy to provide at that point. But, if it's well proven at that point then I don't have a problem.” (ID 25, F, 40) |
| **Possible AI outcomes** | Potential benefits and limitations of AI | “There’s just some things that the machines are very much better at than humans, so in some ways the machine has many great advantages.” (ID 80, M, 65) |
| **Training needed for staff with AI** | Need for staff to have appropriate training for using AI | “There needs to be people running the AI and so, that [safety] would have to be brought up in training for them.” (ID 55, F, 65) |
| **Standardized approach** | Need for standardized practices, decision making, and processes when using AI in healthcare | “They would also need to let me know if the biopsy is being performed during the same session as the MRI and that there are standardized procedures in place to ensure accuracy. I would not want it to be a decision made by the AI itself.” (ID 30, M, 62) |
